# Does endometrial morular metaplasia represent odontogenic differentiation?

**DOI:** 10.1007/s00428-021-03060-2

**Published:** 2021-03-05

**Authors:** Antonio Travaglino, Antonio Raffone, Daniela Russo, Elia Guadagno, Sara Pignatiello, Paola Moretta, Fulvio Zullo, Marialaura Del Basso De Caro, Luigi Insabato, Massimo Mascolo

**Affiliations:** 1 Pathology Unit, Department of Advanced Biomedical Sciences, University of Naples “Federico II”, Naples, Italy; 2 Gynecology and Obstetrics Unit, Department of Neurosciences, Reproductive Sciences and Dentistry, University of Naples “Federico II”, Naples, Italy

**Keywords:** Endometrial, Endometrioid, Morula, Squamous, CTNNB1

## Abstract

The nature of endometrial morular metaplasia (MorM) is still unknown. The nuclear β-catenin accumulation and the not rare ghost cell keratinization suggest a similarity with hard keratin-producing odontogenic and hair matrix tumors rather than with squamous differentiation. We aimed to compare MorM to hard keratin-producing tumors. Forty-one hard keratin-producing tumors, including 26 hair matrix tumors (20 pilomatrixomas and 6 pilomatrix carcinomas) and 15 odontogenic tumors (adamantinomatous craniopharyngiomas), were compared to 15 endometrioid carcinomas with MorM with or without squamous/keratinizing features. Immunohistochemistry for β-catenin, CD10, CDX2, ki67, p63, CK5/6, CK7, CK8/18, CK19, and pan-hard keratin was performed; 10 cases of endometrioid carcinomas with conventional squamous differentiation were used as controls. In adamantinomatous craniopharyngiomas, the β-catenin-accumulating cell clusters (whorl-like structures) were morphologically similar to MorM (round syncytial aggregates of bland cells with round-to-spindled nuclei and profuse cytoplasm), with overlapping squamous/keratinizing features (clear cells with prominent membrane, rounded squamous formations, ghost cells). Both MorM and whorl-like structures consistently showed positivity for CD10 and CDX2, with low ki67; cytokeratins pattern was also overlapping, although more variable. Hard keratin was focally/multifocally positive in 8 MorM cases and focally in one conventional squamous differentiation case. Hair matrix tumors showed no morphological or immunophenotypical overlap with MorM. MorM shows wide morphological and immunophenotypical overlap with the whorl-like structures of adamantinomatous craniopharyngiomas, which are analogous to enamel knots of tooth development. This suggests that MorM might be an aberrant mimic of odontogenic differentiation.

**Supplementary Information:**

The online version contains supplementary material available at 10.1007/s00428-021-03060-2.

## Introduction

Morular metaplasia (MorM) is a common type of altered differentiation found in endometrioid lesions [1]. The nature of MorM has not yet been defined: several authors support that it is an immature form of squamous differentiation (SqD), while others consider it a completely different entity [1–8]. Morphologically, MorM has been defined by the typical syncytial appearance with bland nuclei and profuse cytoplasm. The lack of overt squamous features, such as prominent cell membranes and keratinization, has been used to distinguish MorM from conventional SqD [8]. However, we and others highlighted that MorM not uncommonly displays overt squamous features and a characteristic ghost cell keratinization [9–11]. Immunophenotypically, MorM differs from conventional SqD. Squamous cell markers, such as p40, p63, and cytokeratin (CK) 5/6, can be expressed in MorM, although less commonly and intensely than conventional SqD. Instead, the expression of β-catenin (nuclear, due to *CTNNB1* mutation), CD10, and CDX2 appears as a hallmark of MorM. Unlike SqD, MorM typically shows decreased CK7 expression compared to the background endometrium; CK8/18 expression, typically strong in both MorM and conventional SqD, may be lost in squamous cells originating from MorM [5, 6, 8, 9, 12–15].

Based on the presence of nuclear β-catenin accumulation and the ghost cell keratinization, it has been suggested that MorM might represent a differentiation towards hair [10]. In fact, tumors originating from the hair matrix, such as pilomatrixoma and pilomatrix carcinoma, often show nuclear β-catenin expression (due to *CTNNB1* mutation) and ghost cell keratinization (due to the presence of hard keratin). However, the described features are also present in tumors exhibiting odontogenic differentiation, such as calcifying odontogenic cyst and adamantinomatous craniopharyngioma (ACP) [16, 17]. In these tumors, the presence of ghost cells and hard keratin seems to represent an irregular deposition of tooth enamel matrix rather than aberrant hair formation [18, 19]. In spite of these similarities, a systematic comparison between MorM and hard keratin-expressing tumors has never been performed. Furthermore, to our knowledge, hard keratins have never been assessed in MorM. The aim of this study was to assess whether MorM may represent a differentiation towards hair or tooth, by comparing MorM to hard keratin-expressing tumors.

## Materials and methods

### Case selection

All specimens were retrieved from the archives of the Pathology Unit of the Federico II University Polyclinic of Naples. The study sample included 15 endometrioid carcinomas with MorM (out of which 11 showed overt squamous/keratinizing features) diagnosed between January 2019 and October 2020; 10 of these cases were previously described [9]. Twenty pilomatrixomas diagnosed in the 2019–2020 period and 6 pilomatrix carcinomas diagnosed in the 2010–2020 period were selected as tumors representative of hair matrix differentiation. Fifteen cases of adamantinomatous craniopharyngioma (ACP) diagnosed in the 2003–2019 were also selected as tumors representative of odontogenic differentiation; 11 out of 15 ACP were previously reported [20]. Ten previously described cases of endometrioid carcinoma with conventional SqD were used as controls [9].

### Histological and immunohistochemical methods

Histological methods for endometrioid carcinomas were previously reported [9]. For pilomatrixomas and pilomatrix carcinomas, the lesion was sampled in toto and surgical margins were assessed separately. Calcifying odontogenic cysts and ACP were sampled in toto. All samples were fixed in formalin for 24–48h. Tissue processing procedures were the same for all samples. Immunohistochemistry for β-catenin, CD10, CDX2, p63, ki67, CK5/6, CK7, CK8/18, and CK19 was performed following previously described methods [9]. Immunohistochemistry for pan-hard keratin was carried out by using anti-type I+II hair keratins antibody (guinea pig polyclonal antibody; 1:100), produced by PROGEN®, Maaßstraße 30, 69123 Heidelberg, Germania (cat. no. GP-PANHK); the antibody recognizes human hard keratins of both type I (K31–K40) type II (K81–K86). The procedure was performed by using an automatized platform (VENTANA BenchMark XT®, Roche, Basel, Switzerland), through the following steps: deparaffination, antigen retrieval (98°C, pH 9, 30 m), inhibition of endogenous peroxidase, incubation with primary antibody (1 h, dilution 1:100, 37°C), addition of a polymeric system to amplify the signal (10 m), addition of the secondary antibody conjugated to peroxidase (20 m), addition of diaminobenzidine+substrate (10 m), nuclear counterstaining with hematoxylin. Immunohistochemical methods and antibodies used are summarized in Table 1.

**Table 1:** Antibodies and methods used for immunohistochemical analyses

Marker	Antibody		Dilution	Antigen Retrieval	Incubation	Manufacturer
	Clone	Species				
β-catenin	14	Mouse	Prediluted (2.3μg/ml)	98°C, pH 6, 30m	37°C, 32m	Cell Marque
CD10	SP67	Rabbit	Prediluted (4.9μg/ml)	98°C, pH 6, 90m	37°C, 28m	Ventana
CDX2	EPR2764Y	Rabbit	Prediluted (0.21μg/ml)	98°C, pH 6, 30m	37°C, 32m	Cell Marque
Ki67	30-9	Rabbit	Prediluted (2μg/ml)	98°C, pH 6, 30m	37°C, 48m	Ventana
P63	4A4	Mouse	Prediluted (0.14μg/ml)	98°C, pH 6, 30m	37°C, 32m	Ventana
Cytokeratin 5/6	D5/16B4	Mouse	Prediluted (10.4μg/ml)	98°C, pH 6, 30m	37°C, 48m	Ventana
Cytokeratin 7	SP52	Rabbit	Prediluted (0.536μg/ml)	98°C, pH 6, 30m	37°C, 32m	Ventana
Cytokeratin 8/18	B22.1&B23.1	Mouse	Prediluted (1.56μg/ml)	98°C, pH 6, 30m	37°C, 32m	Cell Marque
Cytokeratin 19	A53-B/A2.26	Mouse	Prediluted (0.11μg/ml)	98°C, pH6, 30m	37°C, 32m	Cell Marque
Pan-hard keratin	Polyclonal	Guinea pig	1:100	98°C, pH9, 30m	37°C, 60m	Progen

### Pathological evaluation

All cases underwent complete immunohistochemical assessment. ACP cases were used as positive controls for hard keratin based on published data [17], while glandular epithelium and epidermis served as internal negative controls in endometrioid carcinomas and hair matrix tumors, respectively. For the other markers assessed, representative cases of endometrioid carcinomas with MorM and conventional SqD from our previous study [9] were used as controls, since the presence of different tumoral components (glands, MorM, squamous features within MorM, conventional SqD) offered both positive and negative controls. Hair matrix and odontogenic tumors were screened by immunohistochemistry for β-catenin, in order to identify tumoral areas suitable for comparison with MorM. Cases were considered relevant if they exhibited (i) distinct nuclear accumulation of β-catenin and (ii) morphological similarity to MorM (i.e., syncytial aggregates of bland cells with round/ovoidal-to-spindled nuclei and profuse cytoplasm) in the same area; squamous/keratinizing features associated with MorM and previously described (i.e., clear cell with distinct cell borders, overtly squamous cells in round aggregates, ghost cells) [9] were also assessed. All specimens were evaluated by one pathologist (AT) and a second subspecialized pathologist at a double-headed microscope (DR, EG, MDBDC, LI, MM). All data were anonymized.

## Results

### Endometrioid carcinomas with MorM

**Fig. 1:**
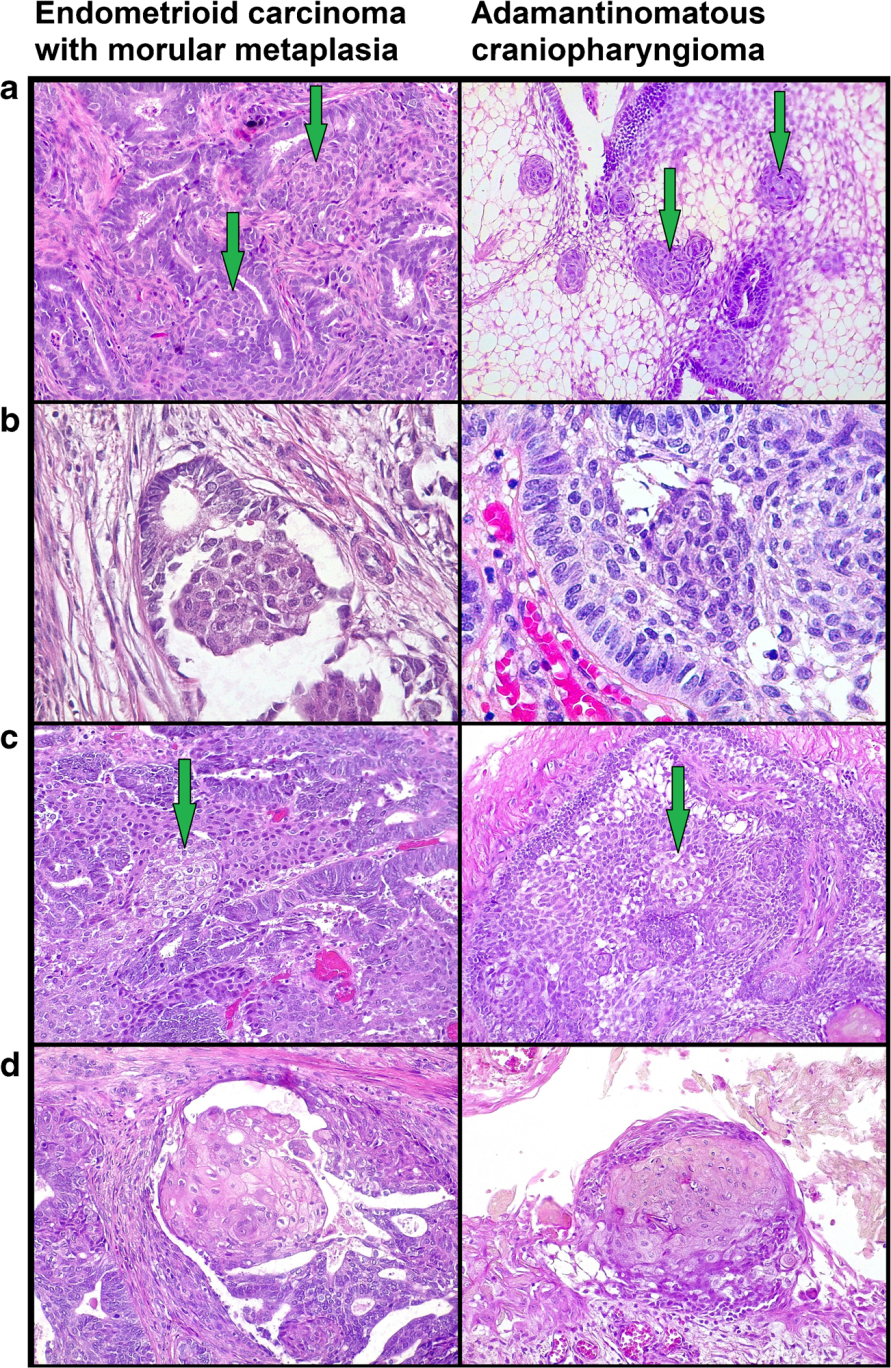
Morphological features of morular metaplasia in endometrioid carcinoma and whorl-like structures in adamantinomatous carcinoma (hematoxylin-eosin). **a** Round shape (magnification ×200). **b** Cytological detail (×400). **c** Clear cells with evident cell membrane (×200). **d** Round aggregates of squamous cells (×200)

**Fig. 2:**
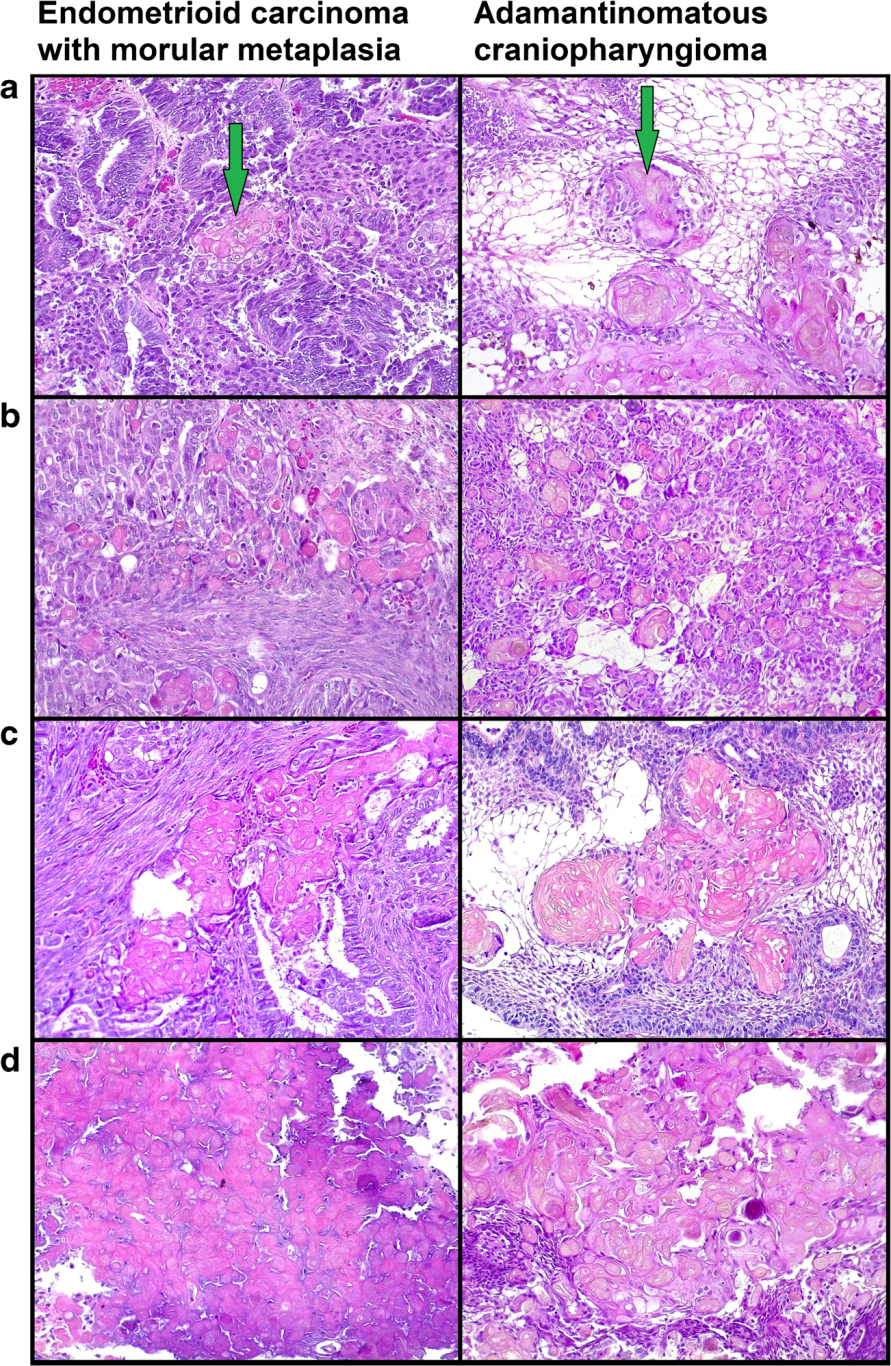
Ghost cell keratinization in endometrioid carcinoma with morular metaplasia and whorl-like structures of adamantinomatous carcinoma (hematoxylin-eosin, magnification ×200). **a** Central ghost cell keratinization surrounded by clear cells. **b** Multiple single ghost cells. **c** Irregular confluent ghost cell keratinization. **d** Diffuse ghost cell keratinization

**Fig. 3:**
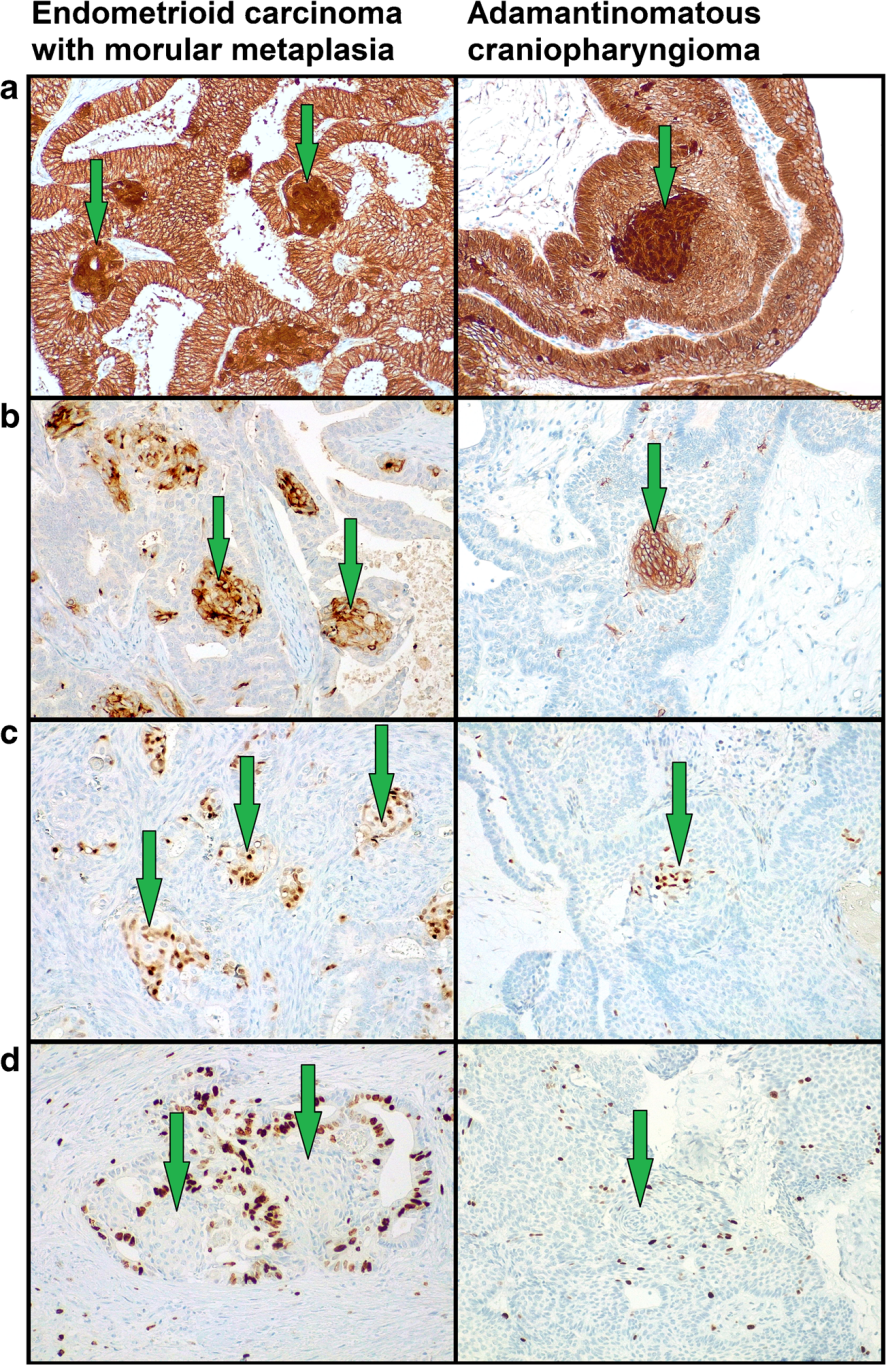
Immunohistochemical features of endometrioid carcinomas with morular metaplasia and whorl-like structures of adamantimomatous craniopharyngioma (magnification ×200X). **a** Nuclear β-catenin accumulation. **b** CD10 positivity. **c** CDX2 positivity. **d** Low/absent ki67 expression

**Fig. 4:**
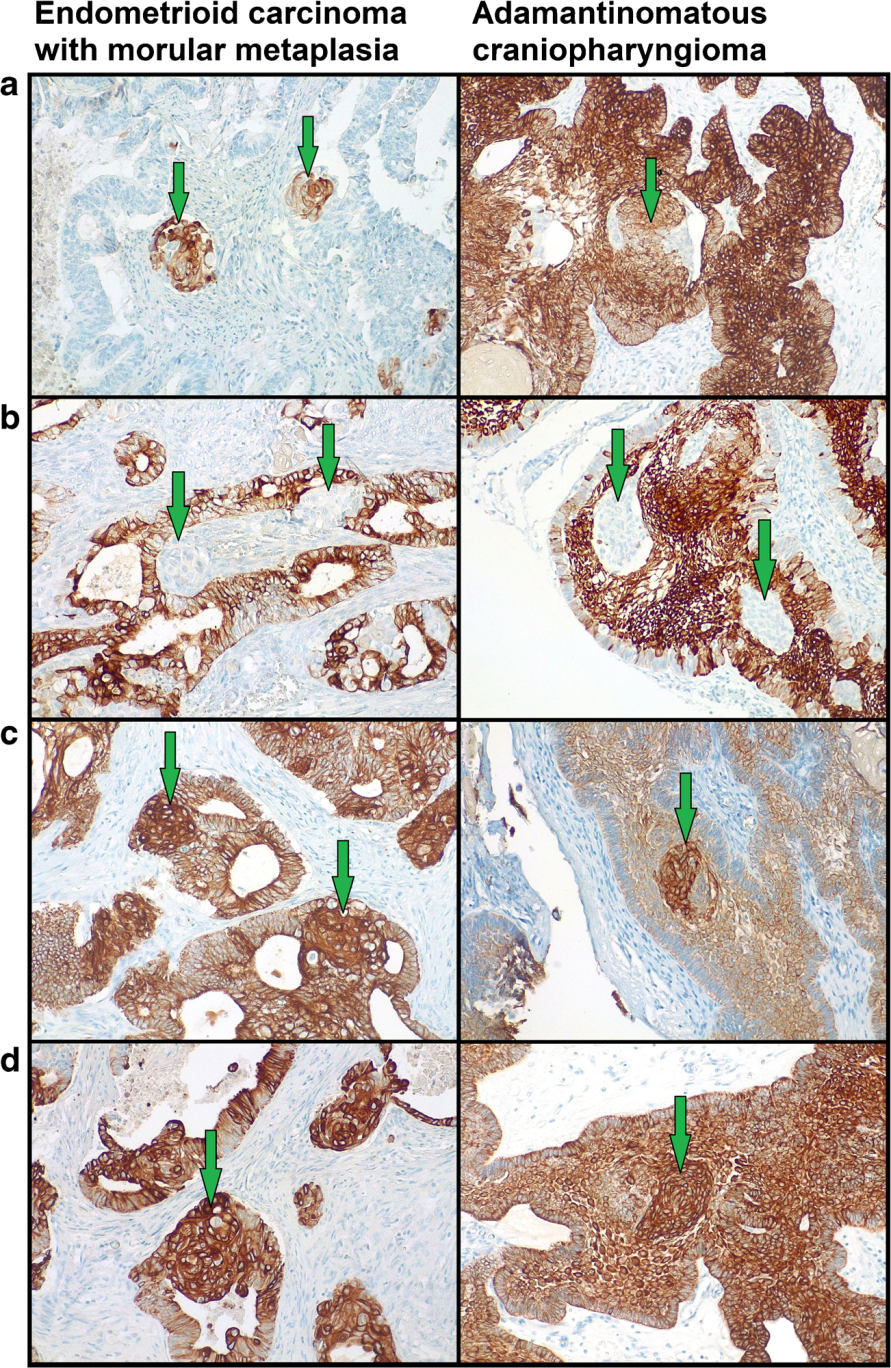
Cytokeratins (CK) expression in endometrioid carcinomas with morular metaplasia and whorl-like structures of adamantimomatous craniopharyngioma (magnification ×200). **a** Variable CK5/6 expression. **b** Decreased CK7 expression compared to the background. **c** Increased CK8/18 expression compared to the background. **d** CK19 positivity similar to the background

**Fig. 5:**
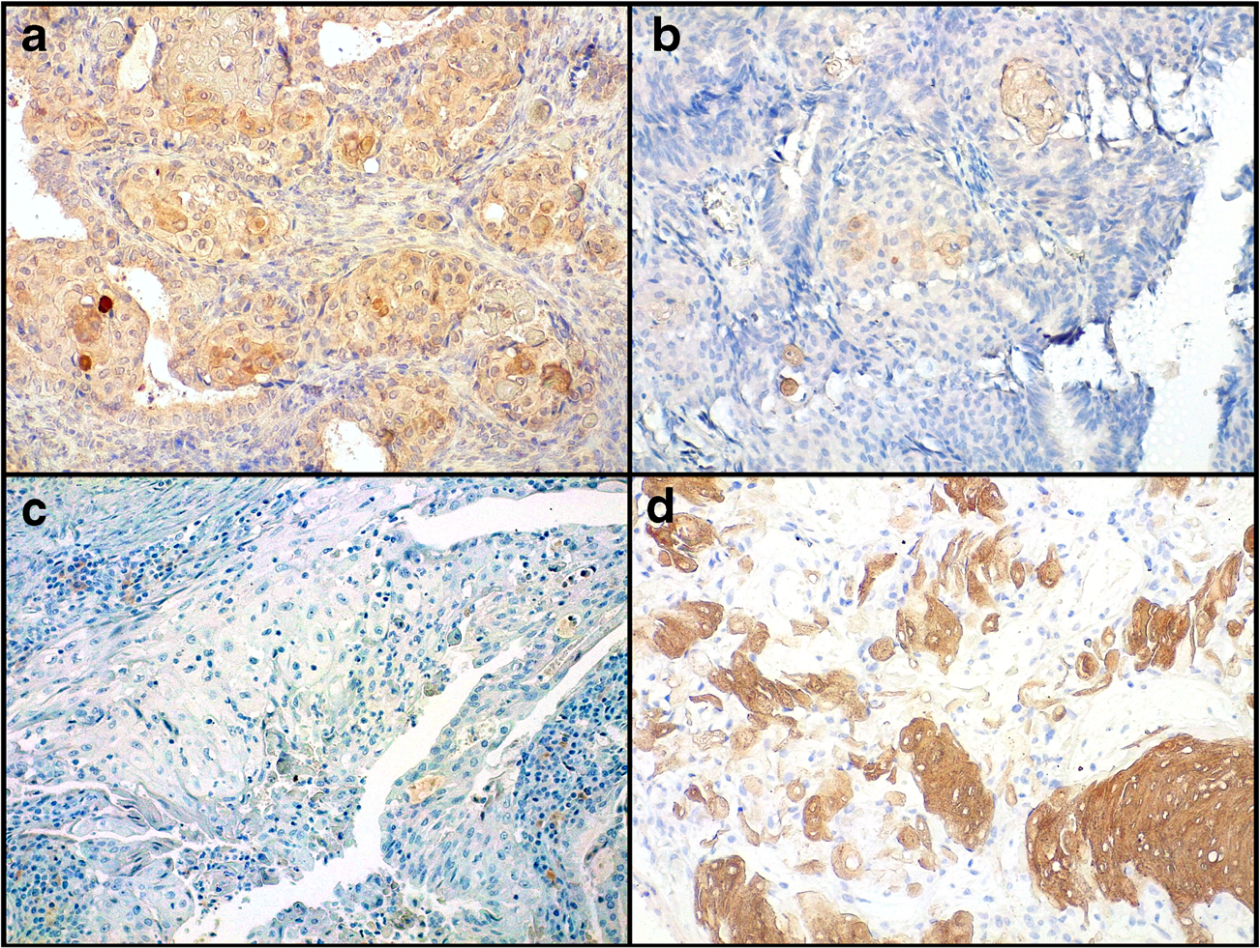
Hard keratin expression (magnification ×200). **a** A case of morular metaplasia with multifocal positivity. **b** A case of morular metaplasia with focal positivity. **c** Negativity in conventional squamous differentiation. **d** Diffuse positivity in the ghost cells of adamantinomatous craniopharyngioma

MorM areas were observed as both rounded formation and irregular sheets (Fig. 1). Eleven out of 15 cases also exhibited squamous/keratinizing features within MorM areas (Figs. 1 and 2). The distribution of squamous/keratinizing features was diffuse in 7 cases and focal in 4 cases. Morphological and immunohistochemical features of 10/15 cases were previously detailed [9]. In brief, all MorM areas consistently showed positivity for β-catenin (nuclear), CD10, and CDX2, with low ki67 expression (Fig. 3); CK8/18 and CK19 positivity, variable CK5/6 positivity, and weak-to-null CK7 expression were observed (Fig. 4). Rudimental squamous features within MorM consisted of clear cell with evident cell borders (Fig. 1) and strong p63 positivity. More mature squamous features consisted of rhomboidal cells with wide eosinophilic cytoplasm and prominent cell membranes, often in a rounded arrangement (Fig. 1); such areas showed loss of the MorM markers (nuclear β-catenin, CD10, and CDX2); p63 expression decreased with the keratinization process [9]. Keratiniziation within MorM appeared either as central ghost cells aggregate or as multiple single ghost cells (Fig. 2). Hard keratin showed multifocal positivity in 3 cases and focal positivity in 5 cases; other 3 cases showed a slight nuance within keratinizing areas which was not considered significant; the 4 MorM cases with no squamous/keratinizing features were negative. In the 10 conventional SqD cases, hard keratin staining was negative in all but one case, which showed focal and weak positivity (Fig. 5).

### Hair matrix tumors

Compared to MorM, the cell nuclei of pilomatrixomas were almost invariably round/ovoidal and regularly spaced, with no spindling. The cytoplasm of basaloid cells was invariably scant, and the squamous cells consistently showed a finely dispersed chromatin. Unlike MorM, the ghost cells of pilomatrixoma were always arranged in tightly cohesive sheets. Out of 17 pilomatrixomas with adequate epithelial component, 13 cases showed nuclear/cytoplasmic β-catenin accumulation in both basaloid and squamous cells. CD10 showed distinct positivity in the basaloid cells, which became weaker in the squamous cells. CDX2 was positive in 13 cases (diffuse in 5 cases and focal/multifocal in 8 cases). Ki67 expression was high in the basaloid cells and low in the squamous cells. P63 was strongly expressed in both components. The CK expression pattern was variable for CK5/6, negative-to-focal for CK7, and CK8/18, and multifocal-to-diffuse for CK19 expression. Pilomatrix carcinomas showed β-catenin accumulation in 3/6 cases; compared to pilomatrixomas, they showed diffuse striking nuclear atypia with high mitotic index and high ki67 expression in all cases. Hard keratin showed distinct expression in the ghost cell layers nearest to the squamous cells in 11 hair matrix tumors; the remaining cases showed weak and focal expression. Morphological and immunophenotypical features of hair matrix tumors are shown in Supplementary Figures 1-3.

### Odontogenic tumors

All ACP showed nuclear β-catenin accumulation in the so-called whorl-like structures (WS). Analogously to MorM, the WS were mostly observed in the form of small, round syncytial aggregates of bland cells, with round/ovoidal-to-spindled nuclei and variable amount of eosinophilic-to-amphophilic cytoplasm (Fig. 1). Cluster of clear cells with evident cell membranes were observed within WS (Fig. 1). Overt squamous features including rhomboidal cells with wide eosinophilic cytoplasm and prominent membranes were also present; these areas often maintained a round, “morular” shape (Fig. 1). Foci of ghost cell keratinization in WS were often associated with clear/squamous cells; in some areas, keratinization occurred in the form of multiple, single ghost cells (Fig. 2).

On immunohistochemistry, WS were sharply distinguished from the background by CD10 and CDX2 positivity, as observed in MorM; ki67 expression was low-to-null (Fig. 3). Compared to the other markers assessed, the expression of CK appeared more heterogeneous, even within the same sample; overall, it was similar to that of MorM: CK5/6 showed variable positivity; CK7 mostly showed decreased expression compared to the surrounding tumoral tissue; CK8/18 showed strong and increased expression compared to the surrounding tissue; CK19 was positive with no appreciable difference from the surrounding tissue (Fig. 4). Unlike MorM, the expression of p63 in WSs was prominent even in the absence of squamous features, resulting similar to the surrounding stellate reticulum; however, analogously to MorM, p63 expression decreased with the development of terminal squamous/keratinizing features. All cases showed diffuse hard keratin expression in the ghost cells, although with variable intensity (Fig. 5).

Similarities and differences among MorM, SqD, WS, and hair matrix tumors are summarized in Table 2.

**Table 2:** Similarities and differences among endometrioid carcinoma with morular metaplasia, endometrioid carcinoma with conventional squamous differentiation, whorl-like structures of adamantinomatous craniopharyngioma, and hair matrix tumors. +, positive; +/−, variable; −/+, negative or focally/weakly positive; −, negative

	Endometrioid carcinoma		Craniopharyngioma	Pilomatrixoma		Pilomatrix carcinoma
	Morular metaplasia	Squamous differentiation	Whorl-like structures	Basaloid cells	Squamous cells	
Arrangement	Rounded	Layered	Rounded	Layered	Layered	Irregular
Nuclear atypia	Bland	Variable	Bland	Bland	Bland	Marked
Nuclear shape	Round/ovoidal to spindled	Irregularly round/ovoidal	Round/ovoidal to spindled	Uniformly round/ovoidal	Uniformly round/ovoidal	Irregularly round/ovoidal
Cytoplasm amount	Abundant	Abundant	Abundant	Scant	Abundant	Abundant
Ghost cells arrangement	Single, in layers or in rounded formations	None	Single, in layers or in rounded formations	None	Tightly cohesive sheets	Variable
Β-catenin (nuclear)	+	−	+	+	+	+/−
CD10	+	−	+	+	+/−	+/−
CDX2	+	−	+	+/−	+/−	+/−
Ki67	Low	Variable	Low	High	Low	High
P63	−/+	+	+	+	+	+
CK5/6	+/−	+	+/−	+/−	+/−	+/−
CK7	−/+	+	−/+	−/+	−/+	−/+
CK8/18	+	+	+	−/+	−	−/+
CK19	+	+	+	+	+	+
Hard keratin	+/−	−	+	−	+	+

## Discussion

This study showed a wide morphological and immunophenotypical overlap between MorM and the WS of ACP; no morphological or immunophenotypical analogies were found between MorM and hair matrix tumors.

The nature of MorM has long since remained a mystery. On the one hand, several features have led to consider MorM as a putative immature form of SqD, e.g., the profuse eosinophilic cytoplasm, the expression of Bcl2 intermediate between glands and squamous areas, the focal presence of tonofilaments on electron microscopy [3, 8, 12]. On the other hand, the unique immunophenotype of MorM (nuclear β-catenin+, CD10+, CDX2+) contrasts with such hypothesis [1, 9, 15]. In addition, some results reported in the literature that might have been misleading in this field. It was reported that MorM showed evidence of neuroectodermal differentiation [7], although such findings have been considered inconclusive due to the inconsistency of the results [21]. Furthermore, several studies separated MorM from conventional SqD based on the absence of overt squamous features and keratinization [5–8, 12, 13]. Due to this view, many cases of conventional SqD with positivity for the MorM markers (nuclear β-catenin, CD10, CDX2) have been reported [5, 12]. As discussed in our previous study, we believe that squamous/keratinizing features are not a criterion to differentiate between MorM and conventional SqD, since it can be observed in both entities. Instead, MorM seems to differ from conventional SqD based on the presence of the typical MorM cellularity with the typical MorM immunophenotype, the variable tendency to maintain a “morular” shape, and the ghost cell keratinization [9]. Therefore, we believe that part of the previously described cases of SqD with MorM immunophenotype, or of mixed MorM/SqD, may represent in fact MorM with squamous/keratinizing features. The evidence of squamous/keratinizing features within MorM appears in agreement with previous observations [10, 11].

The characteristic features of MorM, in particular the nuclear β-catenin accumulation and the ghost cell keratinization, suggest its similarity with hard keratin-producing tumors. Such tumors mainly include hair matrix tumors (i.e., pilomatrixoma and pilomatrix carcinoma) and odontogenic tumors (i.e., calcifying odontogenic cyst and ACP). Analogously to MorM, these tumors harbor *CTNNB1* mutation (which underlies the nuclear β-catenin accumulation); the ghost cell is related to the presence of hard keratin [16, 17]. The analogy between MorM and hard keratin-producing tumors has previously been postulated by Tanaka, who supported that MorM could represent a differentiation towards hair [10]. However, contrary to it was previously suggested [17], hard keratin in odontogenic tumors seems to represent a component of the tooth enamel and not of hair [18, 19]. In fact, the presence of enamel-related proteins has been shown in the ghost cell of ACP [18]. Nonetheless, to the best of our knowledge, MorM has never been systematically compared to hard keratin-producing tumors.

In the present study, we did not find significant analogies between MorM and hair matrix tumors, since the latter ones showed diffuse nuclear β-catenin accumulation in the absence of the typical MorM cellularity. By assessing ACP, we noticed that the WS showed strong morphological analogies with MorM. Indeed, the rounded shape, the syncytial appearance, the bland round/ovoidal-to-spindled nuclei, and the profuse cytoplasm were identically observed in MorM and WS. The squamous/keratinizing features were also similar, with the presence of round/ovoidal clear cell with prominent membranes, overtly squamous cells in a “morular” arrangement and ghost cell keratinization. We noticed that, while the ghost cells of pilomatrixoma always were densely stratified, those of MorM and ACP often appeared as multiple single, round ghost cells within areas with syncytial cellularity. Although ACP and MorM also show layers of stratified ghost cells, Rumayor et al. showed that these were dyscohesive on electron microscopy, while the ghost cells of pilomatrixoma appeared tightly cohesive [22]; such ultrastructural finding is in agreement with our observation. The immunophenotype of MorM and WS was widely superimposable, with consistent β-catenin (nuclear), CD10, and CDX2 positivity that contrasted with the negative background. Interestingly, CDX2 was previously reported to be negative in ACP [23]. Instead, we found a CDX2 positivity limited to the WS, which could be missed at low magnification. The proliferation marker ki67 was low/absent in both MorM and WS. In endometrioid carcinomas, the low proliferation index of MorM contrasted with the highly proliferating glandular component. In ACP, the proliferation index was also low in the stellate reticulum around WS; however, one of us previously reported that ki67 expression remained low in WS even when the surrounding cells showed increased proliferation [20]. The CK pattern was also similar between MorM and WS, despite being less consistent than β-catenin, CD10, and CDX2; by contrast, hair matrix tumors showed weak/absent expression of CK8/18, which was the most strongly expressed CK in both MorM and WS. The only evident difference was p63, which was positive in MorM only in the presence of overt squamous features, while it was consistently positive in WS. However, such difference might be attributed to the difference between endometrioid carcinoma and ACP, since p63 is negative in the former and positive in the latter. In both endometrioid carcinoma and ACP, positivity for β-catenin (nuclear), CD10, and CDX2 was observed around several ghost cell-keratinizing areas, consistently with their derivation from MorM/WS [9]. In addition to these findings, most MorM cases in our series showed focal/multifocal positivity for hard keratin. Such positivity, although not comparable to that of ACP (which showed diffuse positivity in the ghost cells), was completely absent in all but one SqD case, which showed weak and focal staining. The described findings suggest that MorM of endometrioid carcinomas might be biologically similar to the WS of ACP. Interestingly, a recent study demonstrated that WS are analogous to structures termed “enamel knots,” which play a crucial role in the tooth development [19]. On the account of these findings, we suggest that MorM, just like WS, might be analogous to enamel knots. This would mean that MorM is a form of odontogenic differentiation and the keratinizing process found in MorM is an aberrant mimic of the normal tooth development. Remarkably, while MorM resembles enamel knots, the equivalents of other odontogenic components (such as stellate reticulum) are not found in endometrioid lesions. The reason why endometrium would differentiate into a specific odontogenic structure might lie in the different gene expression among different odontogenic components. In fact, enamel knots differ from the other odontogenic components by nuclear β-catenin accumulation and increased expression of several proteins (such as p21 and ectodysplasin A receptor) [19]. It is possible that endometrioid proliferations share the expression of key molecules with enamel knots; in such scenario, a specific molecular event (e.g., *CTNNB1* mutation) might trigger the development of MorM. This would explain why MorM is considerably more common in endometrium than in other tissues. Further studies are necessary in this field.

## Conclusion

MorM shows nuclear β-catenin expression and not rarely exhibits ghost cell keratinization, as observed in hair matrix and odontogenic tumors. However, we did not find striking morphological or immunophenotypical analogies between MorM and hair matrix tumors. On the other hand, MorM showed considerable morphological and immunophenotypical overlap with the WS of ACP. This may suggest that MorM mimics odontogenic differentiation, in particular the enamel knot of the normal tooth development. Further studies are warranted in this field.

## Supplementary information

Supplementary Figure 1: Cytological detail of hair matrix tumors (magnification 400X). A) Basaloid cells with uniform round/ovoidal nuclei and scant cytoplasm in pilomatrixoma. B) Squamous cells with uniform round/ovoidal nuclei with finely dispersed chromatin in pilomatrixoma. C) Tightly cohesive ghost cells in pilomatrixoma. D) Highly atypical cells with high mitotic index in pilomatrix carcinoma. (PNG 16720 kb)

Supplementary Figure 2: Immunophenotypical features of pilomatrixomas (magnification 200X). A) Nuclear and cytoplasmic β-catenin accumulation. B) CD10 expression in basaloid cells. C) Focal CDX2 expression. D) Diffuse p63 expression. E) High ki67 expression in basaloid cells. F) Hard keratin expression in ghost cells. (PNG 4069 kb)

Supplementary Figure 3: Cytokeratins (CK) expression in pilomatrixoma (magnification 200X). A) CK5/6 positivity. B) Focal CK7 positivity. C) CK8/18 negativity. D) CK19 positivity. (PNG 3762 kb)

## Abbreviations

MorM: Morular metaplasia
SqD: Squamous differentiation
ACP: Adamantinomatous craniopharyngioma
WS: Whorl-like structure

## Declarations

This study was based on retrospective analyses of routine archival formalin-fixed, paraffin-embedded tissue. All patients provided a routine written consent for the use of their biological specimen for research purpose at the time of surgery, in agreement with the Declaration of Helsinki and the indications of Italian Legislative Decrees no. 196/03 and 101/18 (Codex on Privacy). All data were anonymized.
