# Supplementary figures and images for: Does endometrial morular metaplasia represent odontogenic differentiation?
Source: Virchows Arch. 2021 Mar 5;479(3):607–16. doi: 10.1007/s00428-021-03060-2 (PMC8448715; doi:10.1007/s00428-021-03060-2)

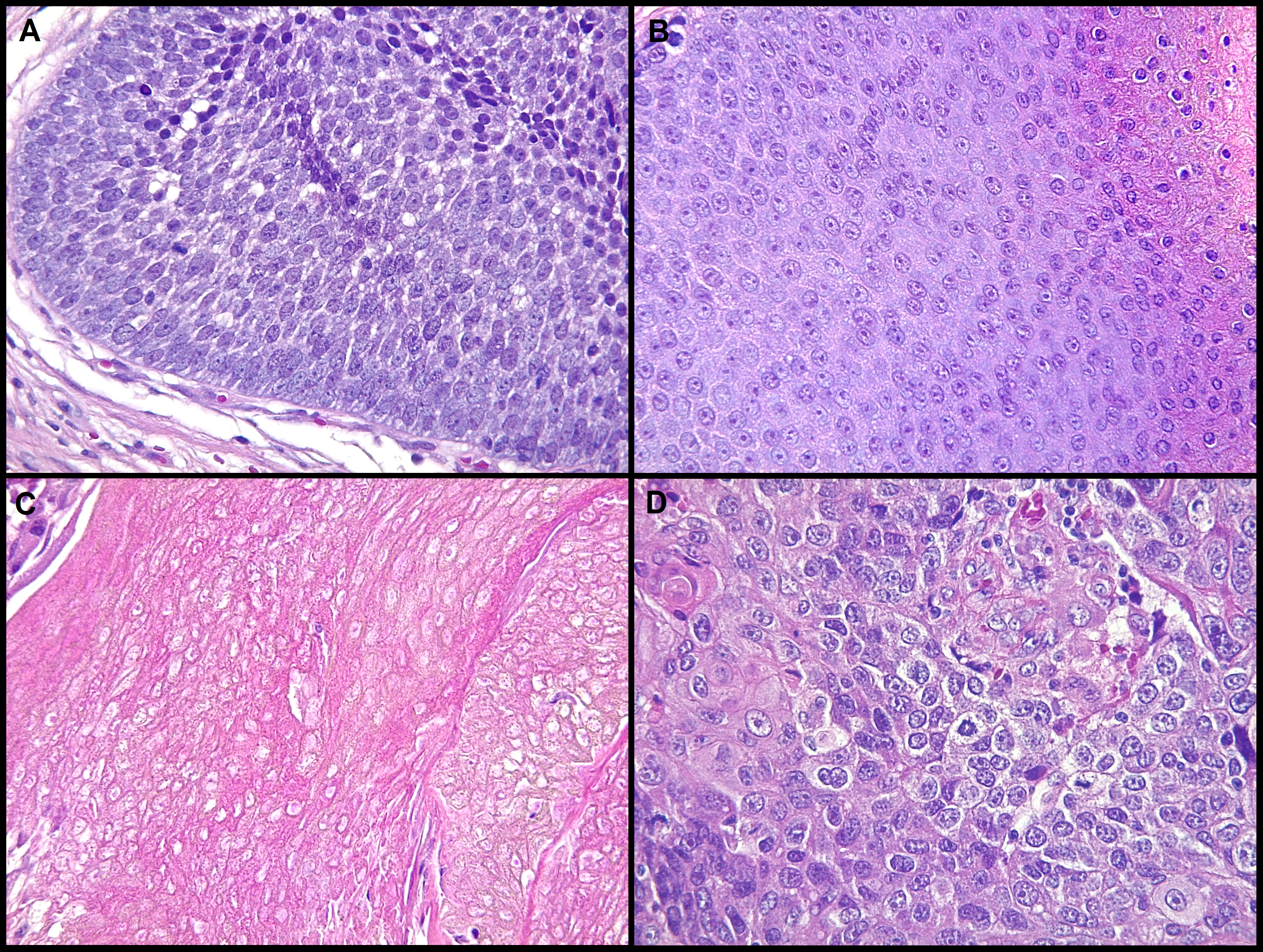

Supplement: Supplementary file 1 — Cytological detail of hair matrix tumors (magnification 400X). A) Basaloid cells with uniform round/ovoidal nuclei and scant cytoplasm in pilomatrixoma. B) Squamous cells with uniform round/ovoidal nuclei with finely dispersed chromatin in pilomatrixoma. C) Tightly cohesive ghost cells in pilomatrixoma. D) Highly atypical cells with high mitotic index in pilomatrix carcinoma. (PNG 16720 kb) [file 428_2021_3060_MOESM1_ESM.png]

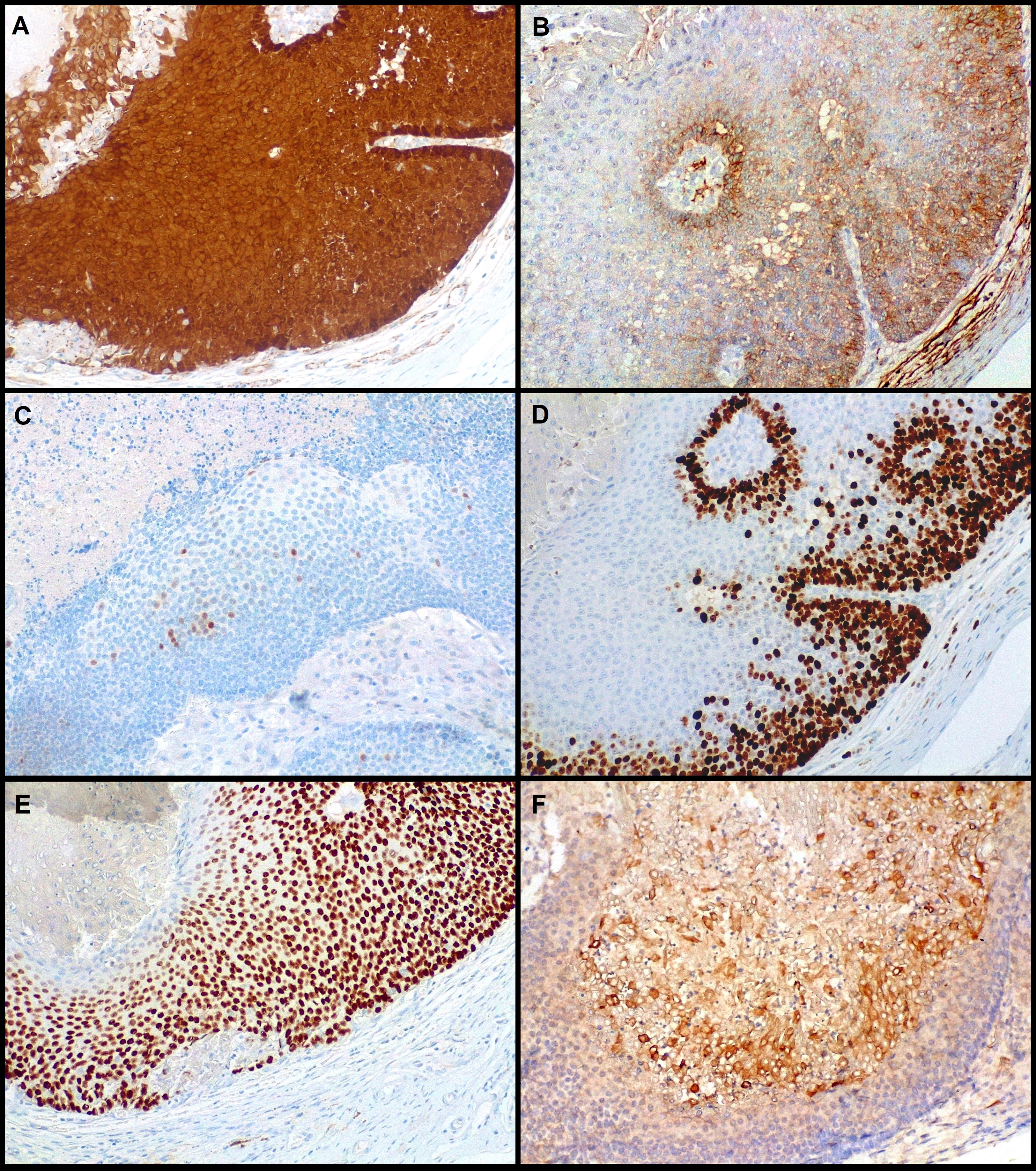

Supplement: Supplementary file 2 — Immunophenotypical features of pilomatrixomas (magnification 200X). A) Nuclear and cytoplasmic β-catenin accumulation. B) CD10 expression in basaloid cells. C) Focal CDX2 expression. D) Diffuse p63 expression. E) High ki67 expression in basaloid cells. F) Hard keratin expression in ghost cells. (PNG 4069 kb) [file 428_2021_3060_MOESM2_ESM.png]

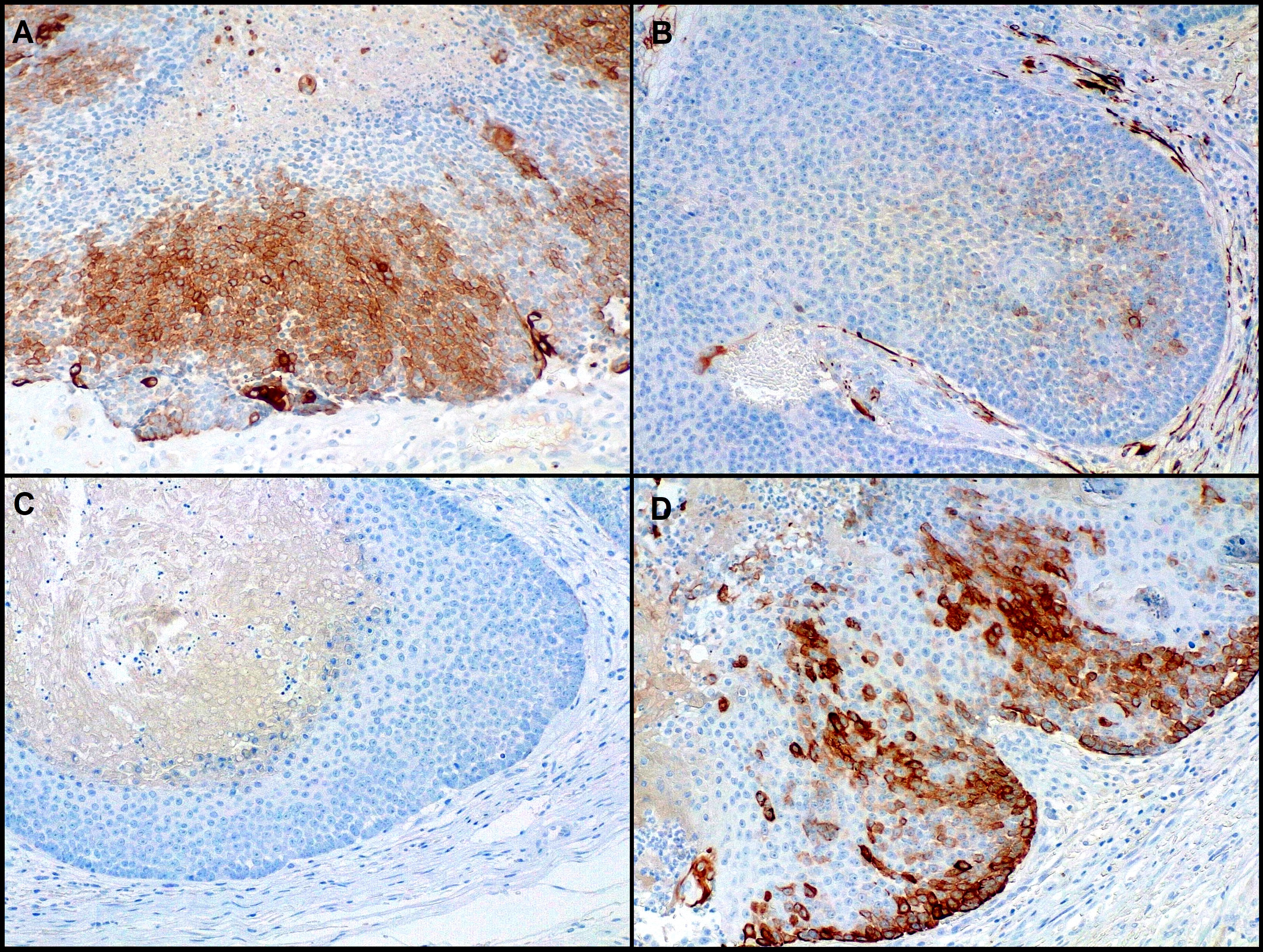

Supplement: Supplementary file 3 — Cytokeratins (CK) expression in pilomatrixoma (magnification 200X). A) CK5/6 positivity. B) Focal CK7 positivity. C) CK8/18 negativity. D) CK19 positivity. (PNG 3762 kb) [file 428_2021_3060_MOESM3_ESM.png]
